# Supplementary material for: Association between antecedent statin use and decreased mortality in hospitalized patients with COVID-19
Source: Nat Commun. 2021 Feb 26;12:1325. doi: 10.1038/s41467-021-21553-1 (PMC7910606; doi:10.1038/s41467-021-21553-1)

SUPPLEMENTAL APPENDIX

Association Between Antecedent Statin Use and Decreased Mortality in Hospitalized Patients with COVID-19

TABLE OF CONTENTS

Page 2	Supplemental Table 1. Mortality rate among patients discharged within 24 hours
Page 3	Supplemental Table 2. ICD-10-CM codes used to classify baseline conditions
Page 4	Supplemental Table 3. Missing rate of laboratory markers
Page 5	Supplemental Figure 1. Distribution of the estimated propensity score for antecedent statin use, among patients who did and did not actually receive the treatment
Page 6	Supplemental Figure 2. Standardized mean differences in the unmatched and matched sample
Page 7	Supplemental Figure 3. Multivariable regression to examine association of antecedent statin use with primary endpoint (in-hospital mortality within 30 days) in study sample restricted to patients with history of hypertension, coronary artery disease and stroke or transient ischemic attack.
Page 8	Supplemental Figure 4. Multivariable regression to examine association of antecedent statin use (modified definition)* with primary endpoint (in-hospital mortality within 30 days)
Page 9	Supplemental Figure 5. Multivariable regression to examine association of inpatient statin use with primary endpoint (in-hospital mortality within 30 days)

Supplemental Table 1. Mortality rate among patients discharged within 24 hours

Among 1508 patients who were in the hospital for less than 24 hours, 267 patients died. Differences in mortality rates by statin use in this excluded population.

	Statin Users	Non-Statin Users
Death	60/397 (15.1%)	207/1111 (18.6%)

Chi-squared p-value = 0.13

Supplemental Table 2. ICD-10-CM codes used to classify baseline conditions

Comorbidity	ICD-10-CM codes
Hypertension	I10-13, I15-16, O10.1-10.4, O10.9
Diabetes	E08 -11, E13, O24.4
Coronary Artery Disease	I21-25, Z98.61, Z95.1
Heart Failure	I09.81, I11.0, I13.0, I50, I42
Chronic Lung Disease	J40-47, J60, J66, J67.2, J67.8-67.9, J68.4, J84, G47.3
Stroke/transient ischemic attack	I60-64, I69, H34.1, G45
Atrial arrhythmias	I47.1, I47.9, I48.0-48.4, I48.91, i48.92, I49.9
Chronic Kidney Disease	N03, N07-08, N11, N14, N18-19, N29, I12, I13, Z99.2, E10.22, E11.22, E13.22, E08.22, O10.3, D63.1
Chronic Liver Disease	K70 – K77

Supplemental Table 3. Missing laboratory markers in the propensity-matched cohort

Laboratory marker	Statin Users (648)	Non-statin Users (648)
White blood cell count	1 (0.1%)	4 (0.6%)
Platelet count	6 (0.9%)	8 (1.2%)
Creatinine	0	0
AST	7 (1.0%)	10 (1.5%)
ALT	11 (1.7%)	12 (1.9%)
Hs-Troponin	34 (5.2%)	47 (7.3%)
Albumin	5 (0.8%)	11 (1.7%)
D-dimer	92 (14.2%)	111 (17.1%)
Ferritin	38 (5.9%)	59 (9.1%)
ESR	80 (12.3%)	74 (11.4%)
CRP	38 (5.9%)	43 (6.6%)

ALT = alanine aminotransferase; AST = aspartate aminotransferase; CRP = C-reactive protein; ESR = erythrocyte sedimentation rate; hs-Troponin = high sensitivity Troponin T; IQR = interquartile range

Supplemental Figure 1. Distribution of the estimated propensity score for antecedent statin use, among patients who did and did not actually receive the treatment

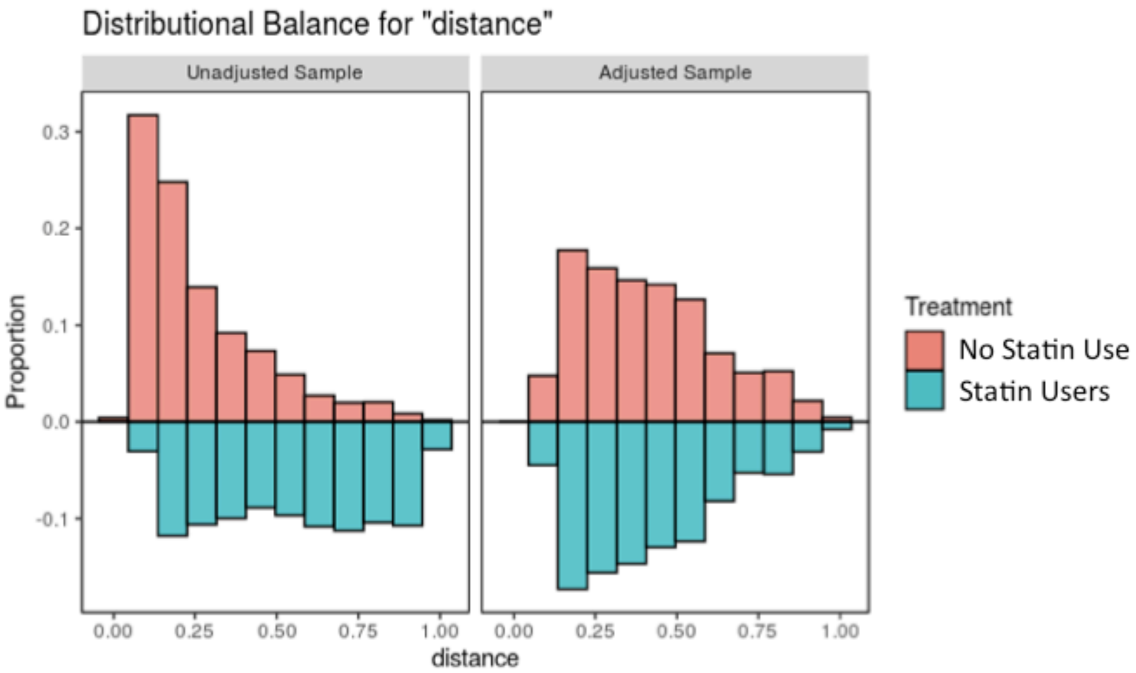

Supplemental Figure 2. Standardized mean differences in the unmatched and matched sample

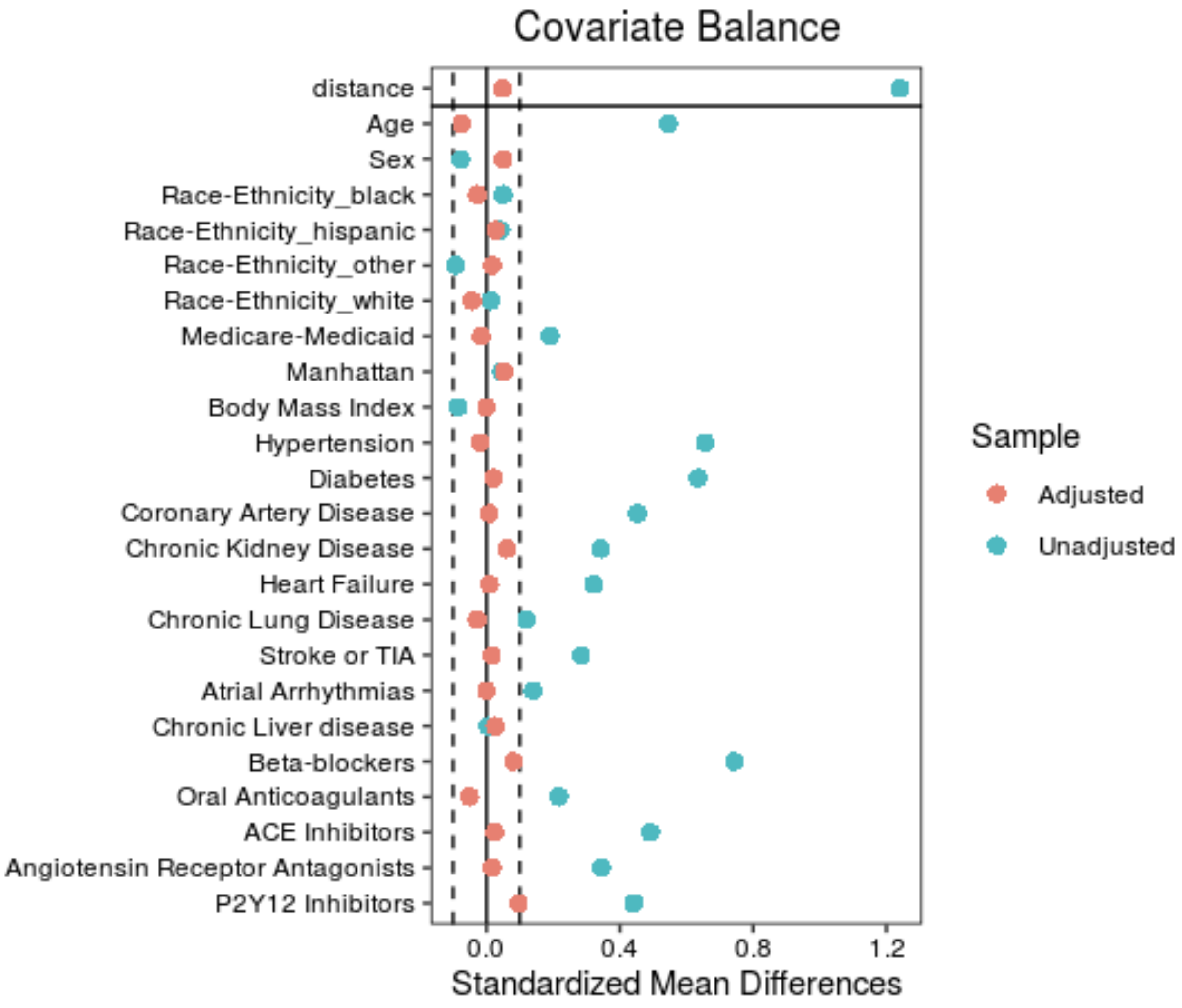

Supplemental Figure 3. Multivariable regression to examine association of antecedent statin use with primary endpoint (in-hospital mortality within 30 days) in study sample restricted to patients with history of hypertension, coronary artery disease and stroke or transient ischemic attack

Forest plot demonstrating the odds ratio (OR) and 95% confidence interval (CI) (N = 1516 independent patients)

	Odds Ratio	Lower CI	Upper CI
Statins	0.438	0.312	0.614
Age (third to first quartile)	4.251	3.049	5.926
Male Sex	1.314	0.958	1.803
Medicare or Medicaid Insurance	0.997	0.718	1.384
Manhattan Residence	1.041	0.764	1.419
BMI (third to first quartile)	1.22	1.014	1.468
Hypertension	1.093	0.543	2.201
Diabetes	1.698	1.239	2.326
Coronary Artery Disease	1.374	0.93	2.03
Chronic Kidney Disease	1.053	0.715	1.55
Heart Failure	1.124	0.715	1.768
Chronic Lung Disease	1.078	0.739	1.572
Stroke	1.139	0.74	1.752
Atrial Arrhythmias	1.699	1.04	2.775
Liver Disease	0.857	0.383	1.917
Beta-Blockers	0.849	0.59	1.221
Oral Anticoagulants	0.377	0.23	0.62
P2Y12 Inhibitors	0.454	0.24	0.86
ACE Inhibitors	1.218	0.757	1.96
Angiotensin Receptor Blockers	1.553	0.98	2.462
Non-Hispanic White	0.742	0.434	1.269
Non-Hispanic Black	1.25	0.772	2.026
Other/missing Race	1.123	0.791	1.594

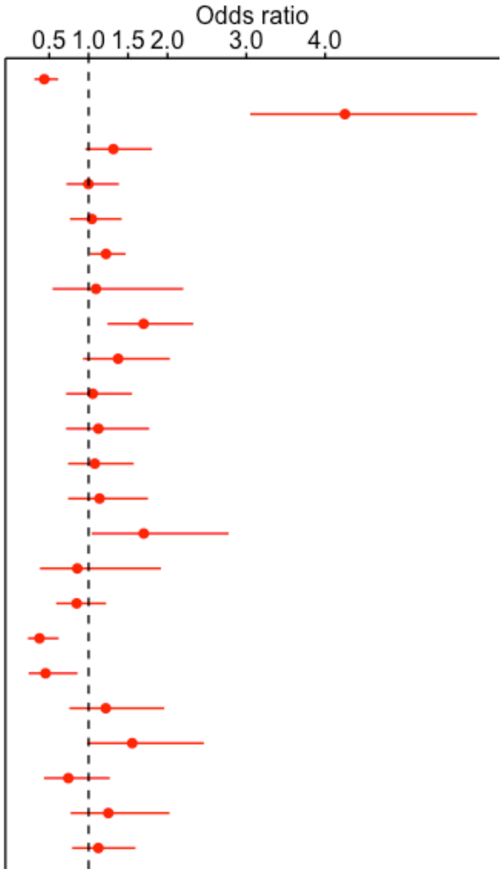

Supplemental Figure 4. Multivariable regression to examine association of statin use (modified definition)* with primary endpoint (in-hospital mortality within 30 days)
Forest plot demonstrating the odds ratio (OR) and 95% confidence interval (CI) (N = 2626 independent patients)

	Odds Ratio	Lower CI	Upper CI
Statins	0.595	0.467	0.758
Age (third to first quartile)	5.072	4.096	6.279
Male Sex	1.46	1.167	1.827
Medicare or Medicaid Insurance	1.131	0.89	1.436
Manhattan Residence	1.047	0.838	1.308
BMI (third to first quartile)	1.043	0.962	1.13
Hypertension	0.818	0.637	1.051
Diabetes	1.35	1.061	1.719
Coronary Artery Disease	1.222	0.875	1.708
Chronic Kidney Disease	1.137	0.827	1.564
Heart Failure	1.391	0.977	1.981
Chronic Lung Disease	1.007	0.754	1.346
Stroke	1.185	0.826	1.701
Atrial Arrhythmias	1.688	1.155	2.468
Liver Disease	0.916	0.491	1.707
Beta-Blockers	0.713	0.533	0.955
Oral Anticoagulants	0.386	0.266	0.562
P2Y12 Inhibitors	0.431	0.247	0.753
ACE Inhibitors	1.151	0.801	1.652
Angiotensin Receptor Blockers	1.317	0.881	1.968
Non-Hispanic White	0.897	0.611	1.316
Non-Hispanic Black	0.968	0.676	1.387
Other/missing Race	1.053	0.821	1.351

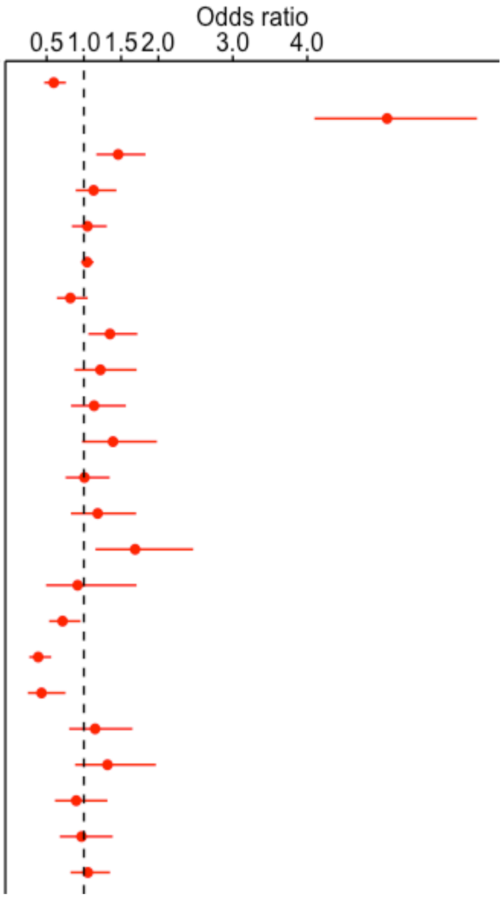

*In this analysis, patients were classified as statin users if they were either prescribed outpatient statin use per the electronic medical record or received statins during hospitalization

Supplemental Figure 5. Multivariable regression to examine association of inpatient statin use with primary endpoint (in-hospital mortality within 30 days)

Forest plot demonstrating the odds ratio (OR) and 95% confidence interval (CI) (N = 2626 independent patients)

	Odds Ratio	Lower CI	Upper CI
Inpatient statins	0.562	0.439	0.721
Age (third to first quartile)	5.03	4.071	6.216
Male Sex	1.467	1.173	1.834
Medicare or Medicaid Insurance	1.131	0.891	1.435
Manhattan Residence	1.035	0.83	1.291
BMI (third to first quartile)	1.049	0.966	1.14
Hypertension	0.821	0.639	1.054
Diabetes	1.342	1.057	1.705
Coronary Artery Disease	1.247	0.892	1.743
Chronic Kidney Disease	1.152	0.838	1.584
Heart Failure	1.415	0.993	2.016
Chronic Lung Disease	0.997	0.747	1.331
Stroke	1.218	0.849	1.745
Atrial Arrhythmias	1.723	1.181	2.514
Liver Disease	0.913	0.49	1.699
Beta-Blockers	0.695	0.52	0.928
Oral Anticoagulants	0.382	0.263	0.555
P2Y12 Inhibitors	0.439	0.251	0.766
ACE Inhibitors	1.089	0.762	1.557
Angiotensin Receptor Blockers	1.28	0.858	1.909
Non-Hispanic White	0.902	0.616	1.322
Non-Hispanic Black	0.962	0.672	1.377
Other/missing Race	1.054	0.822	1.351

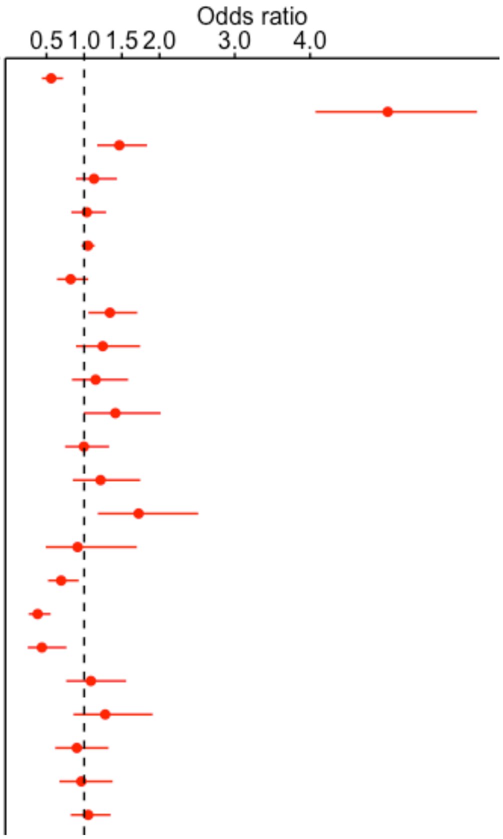

Supplement: Supplementary file 1 — Supplementary Information [file 41467_2021_21553_MOESM1_ESM.pdf]
