# Supplementary material for: Association between antecedent statin use and decreased mortality in hospitalized patients with COVID-19
Source: Nat Commun. 2021 Feb 26;12:1325. doi: 10.1038/s41467-021-21553-1 (PMC7910606; doi:10.1038/s41467-021-21553-1)
Supplement: Supplementary file 3 — Reporting Summary [file 41467_2021_21553_MOESM3_ESM.pdf]

nature research

Corresponding author(s): Aakriti Gupta, MD

Last updated by author(s): Jan 24, 2021

Reporting Summary

Nature Research wishes to improve the reproducibility of the work that we publish. This form provides structure for consistency and transparency in reporting. For further information on Nature Research policies, see our Editorial Policies and the Editorial Policy Checklist.

Statistics

For all statistical analyses, confirm that the following items are present in the figure legend, table legend, main text, or Methods section.

n/a	Confirmed	
☐	☒	The exact sample size (*n*) for each experimental group/condition, given as a discrete number and unit of measurement
☐	☒	A statement on whether measurements were taken from distinct samples or whether the same sample was measured repeatedly
☐	☒	The statistical test(s) used AND whether they are one- or two-sided<br>*Only common tests should be described solely by name; describe more complex techniques in the Methods section.*
☐	☒	A description of all covariates tested
☐	☒	A description of any assumptions or corrections, such as tests of normality and adjustment for multiple comparisons
☐	☒	A full description of the statistical parameters including central tendency (e.g. means) or other basic estimates (e.g. regression coefficient) AND variation (e.g. standard deviation) or associated estimates of uncertainty (e.g. confidence intervals)
☐	☒	For null hypothesis testing, the test statistic (e.g. *F*, *t*, *r*) with confidence intervals, effect sizes, degrees of freedom and *P* value noted<br>*Give P values as exact values whenever suitable.*
☒	☐	For Bayesian analysis, information on the choice of priors and Markov chain Monte Carlo settings
☒	☐	For hierarchical and complex designs, identification of the appropriate level for tests and full reporting of outcomes
☒	☐	Estimates of effect sizes (e.g. Cohen's *d*, Pearson's *r*), indicating how they were calculated

Our web collection on statistics for biologists contains articles on many of the points above.

Software and code

Policy information about availability of computer code

Data collection	No software was used for data collection
Data analysis	R version 3.5.1 was used to analyse the data. We used the packages rms, mice and Matchit.

For manuscripts utilizing custom algorithms or software that are central to the research but not yet described in published literature, software must be made available to editors and reviewers. We strongly encourage code deposition in a community repository (e.g. GitHub). See the Nature Research guidelines for submitting code & software for further information.

Data

Policy information about availability of data

All manuscripts must include a data availability statement. This statement should provide the following information, where applicable:

- Accession codes, unique identifiers, or web links for publicly available datasets
- A list of figures that have associated raw data
- A description of any restrictions on data availability

De-identified data will be made available based on request.

Field-specific reporting

Life sciences study design

All studies must disclose on these points even when the disclosure is negative.

Sample size	The sample size was not estimated, rather all COVID-19 patients who had been hospitalized at our institute with at least a 30 -day follow up period at the time of initiating the analyses were included. Based on our inclusion/exclusion criteria, we narrowed down to 2626 patients, that we thought would be sufficient for the purposes of our analyses.
Data exclusions	We excluded only patients who had a length of stay shorter than 24 hours, as they may not truly represent hospitalizations, but might include ED visits as well.
Replication	To ensure reproducibility, we performed 3 sensitivity analyses for the primary endpoint, that we also included in our supplement. The results of all of these are in agreement. In addition to propensity matching, we also performed multivariable logistic regression on the overall cohort. We also applied a modified definition of statin use and also narrowed the cohort to only those with cardiovascular disease. In all of these anlayses, the effect size and confidence intervals remained similar.
Randomization	As this is an observatinal study, our data was not randomized. However, we performed propensity matching to decrease confounding as much as possible. This has been described in out methods. We also performed multivariable logistic regression on the overall cohort to evaluate for consistency of results.
Blinding	The investigators were not blinded to the groups at the time of data collection or analysis, as this was an observational study. We think that blinding may not be relevant to our study as our primary outcome is mortality which may not be influenced by bias.

Reporting for specific materials, systems and methods

We require information from authors about some types of materials, experimental systems and methods used in many studies. Here, indicate whether each material, system or method listed is relevant to your study. If you are not sure if a list item applies to your research, read the appropriate section before selecting a response.

Materials & experimental systems

n/a	Involved in the study	
☒	☐	Antibodies
☒	☐	Eukaryotic cell lines
☒	☐	Palaeontology and archaeology
☒	☐	Animals and other organisms
☐	☒	Human research participants
☐	☒	Clinical data
☒	☐	Dual use research of concern

Methods

n/a	Involved in the study	
☒	☐	ChIP-seq
☒	☐	Flow cytometry
☒	☐	MRI-based neuroimaging

Human research participants

Policy information about studies involving human research participants

Population characteristics	We have described the population characteristics in the Results section of our manuscript. In general, of 2626 patients included in the analysis, 951 (36.2%) were antecedent statin users. On average, patients who were prescribed statins were older ([median 70 (IQR 63-79) vs. 62 (49-76) years, p<0.001] with no significant differences in sex (p=0.06) or race/ethnicity (p=0.12). Furthermore, patients using statins were significantly more likely to have hypertension (74.0% vs. 43.3%), diabetes (55.8% vs. 26.1%), coronary artery disease (22.5% vs. 6.9%), heart failure (17.0% vs. 6.7%), and chronic kidney disease (22.0% vs. 9.6%) compared with patients not receiving statins (p<0.001 for all). Similarly, patients receiving statins had higher rates of history of stroke/transient ischemic attack (13.9% vs. 5.6%) and atrial arrhythmias (11.0% vs. 5.6%), p<0.001 for both.
Recruitment	We included all patients hospitalized with COVID-19 at our institute during the study period, and performed a retrospective study of their medical records.
Ethics oversight	The Columbia University Irving Medical Center Institutional Review Board approved this study and waived the requirement for obtaining informed consent.

Note that full information on the approval of the study protocol must also be provided in the manuscript.

Clinical data

Policy information about clinical studies

All manuscripts should comply with the ICMJEguidelines for publication of clinical research and a completedCONSORT checklist must be included with all submissions.

Clinical trial registration	This study was not registered with clinicaltrials.gov. This is an observational study performed from our instoture's electronic medical record to help disseminate knowledge gained from our experience with COVID-19.

Study protocol	This study was performed from data relating to COVID-19 hospitalizations from our hospital. We submitted a protocol to COlumbia University's IRB, but that is not publicly available.
Data collection	We collected data on patients hospitalized with COVID-19 from February 1st through May 12th with follow-up till June 11th. These data were abstracted from our hospital's electronic medical record.
Outcomes	We pre-defined our primary outcome to be in-hospital mortality as recorded in our electronic medical record. Our secondary outcome was invasive mechanical ventilation at 30 days revised according to reviewer comments, also abstracted from our electronic medical record.
